# Comprehensive comparative analysis of prognostic value of serum systemic inflammation biomarkers for colorectal cancer: Results from a large multicenter collaboration

**DOI:** 10.3389/fimmu.2022.1092498

**Published:** 2023-01-05

**Authors:** Hailun Xie, Guotian Ruan, Lishuang Wei, Heyang Zhang, Yizhong Ge, Qi Zhang, Mengmeng Song, Xi Zhang, Xiaoyue Liu, Shiqi Lin, Ming Yang, Chunlei Hu, Meng Tang, Li Deng, Wen Hu, Hanping Shi

**Affiliations:** ^1^ Department of Gastrointestinal Surgery/Department of Clinical Nutrition, Beijing Shijitan Hospital, Capital Medical University, Beijing, China; ^2^ Beijing International Science and Technology Cooperation Base for Cancer Metabolism and Nutrition, Beijing, China; ^3^ Key Laboratory of Cancer FSMP for State Market Regulation, Beijing, China; ^4^ Department of Geriatric Respiratory Medicine, The First Affiliated Hospital, Guangxi Medical University, Nanning, Guangxi, China; ^5^ Clinical Nutrition Department, Sichuan University West China Hospital, Chengdu, Sichuan, China

**Keywords:** systemic inflammation, inflammatic burden Index, colorectal cancer, prognosis, biomarker, malnutrition

## Abstract

**Background:**

The incidence of colorectal cancer (CRC) is common and reliable biomarkers are lacking. We aimed to systematically and comprehensively compare the ability of various combinations of serum inflammatory signatures to predict the prognosis of CRC. Moreover, particular attention has been paid to the clinical feasibility of the newly developed inflammatory burden index (IBI) as a prognostic biomarker for CRC.

**Methods:**

The discrimination capacity of the biomarkers was compared using receiver operating characteristic curves and Harrell’s C-index. Kaplan-Meier curves and log-rank tests were used to compare survival differences between the groups. Cox proportional hazard regression analysis was used to determine the independent prognostic factors. Logistic regression analysis was used to assess the relationship between IBI, short-term outcomes, and malnutrition.

**Results:**

IBI had the optimal prediction accuracy among the systemic inflammation biomarkers for predicting the prognosis of CRC. Taking IBI as a reference, none of the remaining systemic inflammation biomarkers showed a gain. Patients with high IBI had significantly worse overall survival than those with low IBI (56.7% vs. 80.2%; log-rank P<0.001). Multivariate Cox regression analysis showed that continuous IBI was an independent risk factor for the prognosis of CRC patients (hazard ratio = 1.165, 95% confidence interval [CI] = 1.043–1.302, P<0.001). High IBI was an independent risk factor for short-term outcomes (odds ratio [OR] = 1.537, 95% CI = 1.258–1.878, P<0.001), malnutrition (OR = 2.996, 95% CI = 1.471–6.103, P=0.003), and recurrence (OR = 1.744, 95% CI = 1.176–2.587, p = 0.006) in CRC patients.

**Conclusions:**

IBI, as a reflection of systemic inflammation, is a feasible and promising biomarker for assessing the prognosis of CRC patients.

## Introduction

Cancer remains one of the leading causes of death worldwide and a major obstacle to improving life expectancy in the 21st century. Colorectal cancer (CRC) is one of the most common cancers and ranks third in both morbidity and mortality among men and women, seriously threatening human health (1). In China, CRC is the second most common cancer and the fourth leading cause of cancer-related deaths. The burden of cancer is expected to increase over the next decade as the population ages (2). Despite continuous improvements in surgery and adjuvant chemoradiotherapy, the recurrence and mortality rates of CRC remain high (3, 4). Therefore, there is an urgent need to identify useful biomarkers that can predict the prognosis of CRC in clinical practice to better formulate the prevention, screening, and treatment for patients with CRC.

As the most representative host-tumor interaction, systemic inflammation is considered to be the seventh hallmark of cancer. Recently, there has been increasing evidence that systemic inflammation is closely related to cancer genesis, cancer progression (invasion, migration, and metastasis), and treatment response (5, 6). It is reported that immunotherapy for solid tumor patients with a high proportion of inflammatory cells in the tumor microenvironment or a tumor mutational burden can effectively improve the prognosis (7). Systemic inflammation is mediated by cytokines and inflammatory cells, which constitute the tumor microenvironment suitable for tumor cell survival. These inflammatory mediators can be detected in routine blood tests in peripheral blood, including blood cells or inflammation-related proteins (8). These blood characteristics and their combinations have been widely reported as effective biomarkers for predicting clinical outcomes in CRC patients (9–11). Okugawa et al. (9) summarized the combination of peripheral systemic inflammatory signatures and compared their value in predicting the prognosis in CRC patients. Yamamoto et al (11). summarized the effect of systemic inflammation biomarkers on the prognosis of CRC patients in 2021. However, certain limitations exist due to the limited sample size and failure to systematically and comprehensively compare the clinical feasibility of current systemic inflammation biomarkers. It is still unclear which combination of blood characteristics is the strongest predictor of prognosis in CRC patients.

Recently, our team developed a novel systemic inflammation biomarker called the inflammatory burden index (IBI), which can be effectively used to predict the prognosis of cancer (12). The performance of IBI in predicting the prognosis of CRC patients is unclear. Therefore, this study aimed to systematically and comprehensively compare the ability of various combinations of inflammatory signatures to predict the prognosis of CRC. Moreover, particular attention has been paid to the clinical feasibility of the newly developed IBI as a prognostic biomarker for CRC.

## Materials and methods

### Study population

This was a prospective multicenter study, with all patients from the Investigation on Nutrition Status and its Clinical Outcome of Common Cancers project, which recruited participants from multiple clinical centers across China, which have been described previously (13, 14). The sample size calculation formula is shown in **Table S1**. The total sample size is 26,300, including 3,780 CRC cases. The cohort has been followed up since 2013. The inclusion criteria were as follows: a) patients with pathologically confirmed CRC, b) patients with a length of hospital stay longer than 48 hours, and c) patients > 18 years of age. The exclusion criteria were as follows: a) incomplete and available peripheral systemic inflammatory signature data, b) patients with severe infection or severe immunodeficiency, and c) admitted to the intensive care unit (ICU) at the beginning of recruitment. This project was registered at http://www.chictr.org.cn (registration number: ChiCTR1800020329). This study was approved by the ethics committees of all the participating institutions. Written informed consent was obtained from all the participating patients.

### Demographic information

Patient demographic information was collected at the time of recruitment, including sex, age, height, weight, comorbidities (hypertension, diabetes), lifestyle (smoking and drinking), family history, tumor-node-metastasis (TNM) stage, anticancer therapy (including surgery, radiotherapy, and chemotherapy, etc.), length of hospital stay, and hospitalization costs. Clinicians conducted a comprehensive interview with each patient to obtain recent anticancer pretreatment nutritional information, including the Karnofsky Performance Scale (KPS) score, Patient-Generated Subjective Global Assessment (PG-SGA), and European Organization for Research and Treatment of Cancer Quality of Life Questionnaire (EORTCQLQ-C30 Version 3.0, Qol). Cancer cachexia was assessed according to the international diagnostic criteria for cancer cachexia (15).

### Laboratory measurements of serum systemic inflammation biomarkers

**Figure 1:**
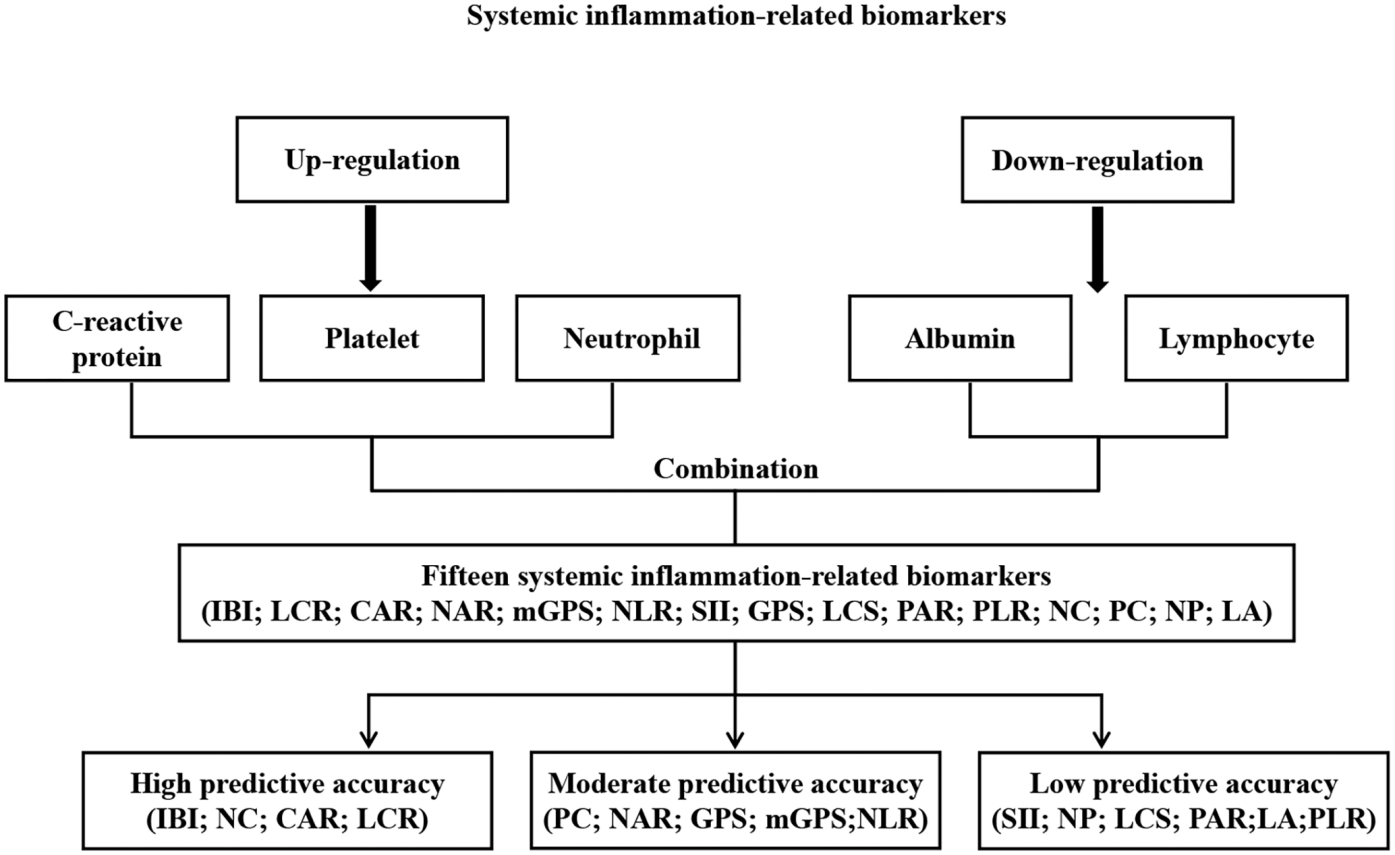
Study design.

Blood samples from each patient were collected within one week before receiving anticancer therapy and then tested at the respective central laboratories for serological information, including counts of whole white blood cells, neutrophils, lymphocytes, platelets, red blood cells, hemoglobin levels, and CRP and albumin levels. Systemic inflammation is characterized by increased proportions of inflammatory parameters (neutrophils, platelets, and C-reactive protein) and decreased proportions of anti-inflammatory parameters (lymphocytes and albumin) (9, 16). Therefore, we combined these five serum inflammatory and anti-inflammatory parameters separately for assessment. Together with the systemic inflammation biomarkers reported in previous studies, 15 biomarkers were identified (**Figure 1**). **Table S2** summarizes the formulae used to calculate these biomarkers. The formula for IBI is defined as C-reactive protein × neutrophil/lymphocyte.

### Outcomes

The primary outcome of this study was overall survival (OS). The secondary outcomes were short-term outcomes and malnutrition status. OS was defined as the interval from cancer diagnosis to death from any cause or last follow-up. Short-term outcomes were defined as the 90-day survival after anticancer therapy. Malnutrition was defined as a PG-SGA score of ≥4. Patients were followed up by professionals from recruitment until death from any cause or the last follow-up (October 30, 2020).

### Statistical analysis

Continuous variables are presented as the mean ±standard deviation or median (interquartile range). Categorical variables are presented as numbers (percentages). The chi-square test or t-test was used to compare differences between the groups. Maximally selected log-rank statistics were used to determine the optimal cutoff values for IBI. The predictive accuracy of the biomarkers was assessed using the receiver operating characteristic curve (ROC). The discrimination capacities of the biomarkers were compared using the Harrell C-index. A restricted cubic spline plot with three knots was used to explore the shape of the correlation between IBI and mortality. Kaplan-Meier curves and log-rank tests were used to compare survival differences between the groups. Cox proportional hazard regression analysis was used to determine the independent prognostic factors. The predicted risk was expressed as a hazard ratio (HR) and 95% confidence interval (CI). Logistic regression analysis was used to assess the relationship between IBI and secondary outcomes. The predicted risk was expressed as an odds ratio (OR) and 95% CI. Two-sided p<0.05 was determined to be statistically significant. All statistical analyses were performed using R version 4.0.5 (http://www.r-project.org).

## Results

### Patient characteristics

Overall, 1296 CRC patients with complete data were included in the study, including 787 men and 509 women. The mean age of the patients was 59.62 (11.77). A total of 113 patients underwent radiotherapy, 907 underwent chemotherapy, and 1154 underwent surgery. A total of 337, 456, and 503 patients were stage I-II, III, and IV, respectively. Sixty-seven patients had adverse short-term outcomes. A total of 408 patients died during the follow-up period. The clinicopathological features of this study are summarized in **Table S3**.

### Comparative analysis of the discrimination of systemic inflammation biomarkers

**Figure 2:**
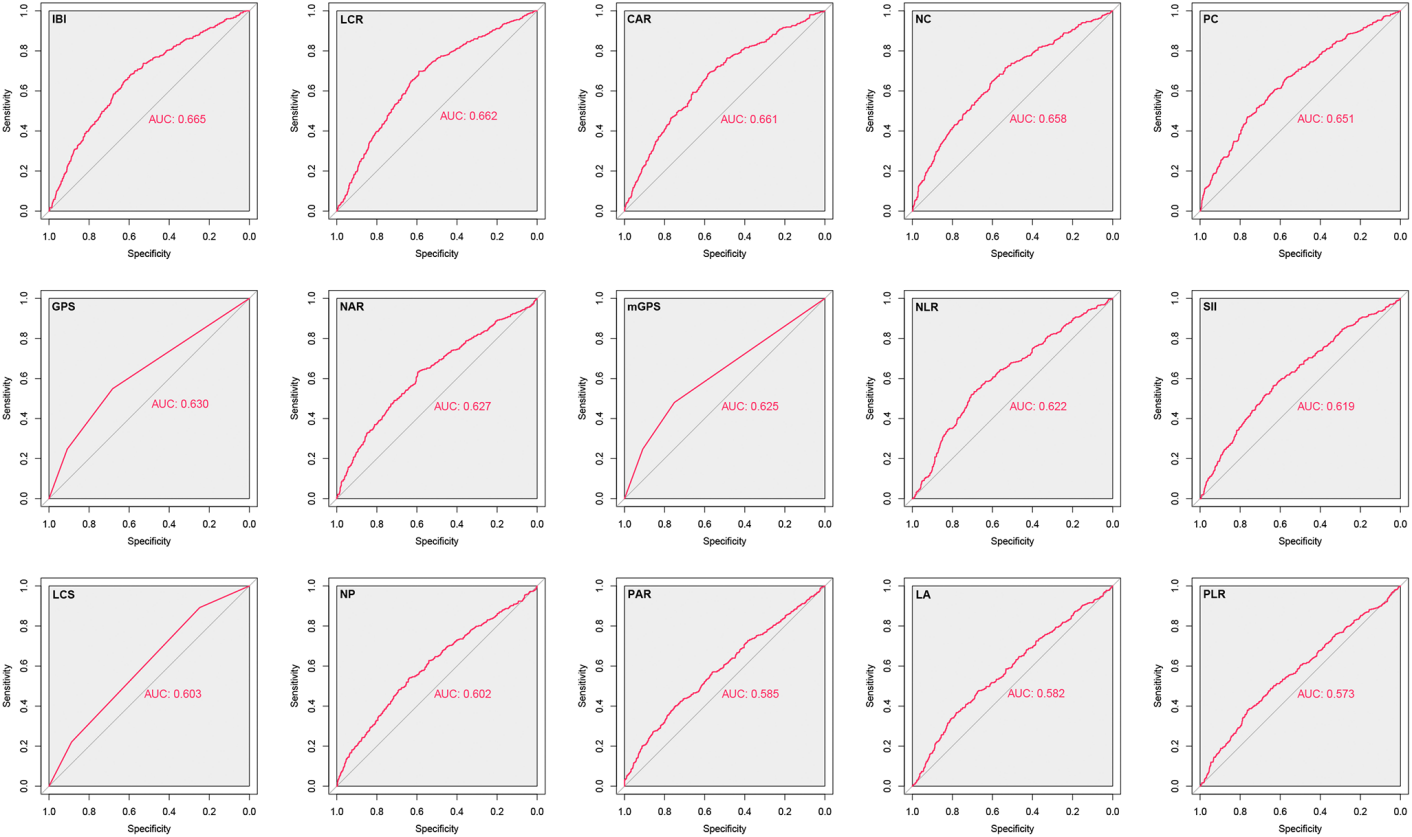
Comparison the effectiveness of systemic inflammation-related biomarkers in predicting the prognosis of CRC patients.

Herein, we found that almost all systemic inflammation biomarkers could significantly predict the prognosis of CRC patients (**Table S4**). We comprehensively analyzed the performance of systemic inflammation biomarkers in predicting the prognosis of CRC patients by ROC analysis. The results showed that IBI had optimal prediction accuracy, and its area under the curve was significantly better than that of other systemic inflammation biomarkers (**Figure 2**). We further found that IBI had the highest Harrell C-index among these systemic inflammation biomarkers for predicting the prognosis of CRC patients, followed by NC, CAR, and LCR (**Table S5**). Taking IBI as a reference, none of the remaining systemic inflammation biomarkers showed a gain. For estimation of mortality risk, each biomarker of systemic inflammation may provide significant incremental prognostic value for TNM stage. The IBI incremental value was statistically significant (**Table S6**). For a more convenient clinical application, we divided them into high/moderate/low predictive accuracy biomarkers according to their ability to predict the prognosis of CRC. High predictive accuracy biomarkers included IBI, NC, CAR, and LCR, with top-ranked predictive ability. Moderate predictive accuracy biomarkers included PC, NAR, GPS, mGPS, and NLR, with moderate predictive ability. Low predictive accuracy biomarkers were defined as biomarkers with a predictive difference greater than 0.05 from IBI, including SII, NP, LCS, PAR, LA, and PLR. The above results suggest that IBI is an optimal systemic inflammation biomarker for assessing the prognosis of CRC patients. Therefore, we subsequently evaluated the potential of IBI as a prognostic predictive biomarker in CRC patients.

### Relationship between IBI and disease development

Based on survival status, we determined that the optimal cutoff value of IBI in CRC patients was 10 (**Figure S1**). A total of 644 CRC patients were classified as having high IBI and 652 CRC patients were classified as having low IBI. Patients with high IBI were closely associated with male sex, advanced age, low body mass index, advanced TNM stage, high comorbidities, adverse lifestyle, high inflammatory status, and low nutritional status. Patients with high IBI tended to have higher hospitalization costs and longer hospital stays (**Table S7**). Furthermore, Spearman’s rank correlations showed that IBI was positively associated with age (men, r=0.051; women, r=0.160), tumor stage (men, r=0.051; women, r=0.140), NRS2002 score (men, r=0.044; women, r=0.060), PG-SGA score (men, r=0.200; women, r=0.210), and EORTC QLQ-C30 score (men, r=0.160; women, r=0.200). IBI was negatively associated with KPS score (men, r=-0.120; women, r=-0.290) (**Figure S2**).

### Survival analysis for dichotomous IBI

**Figure 3:**
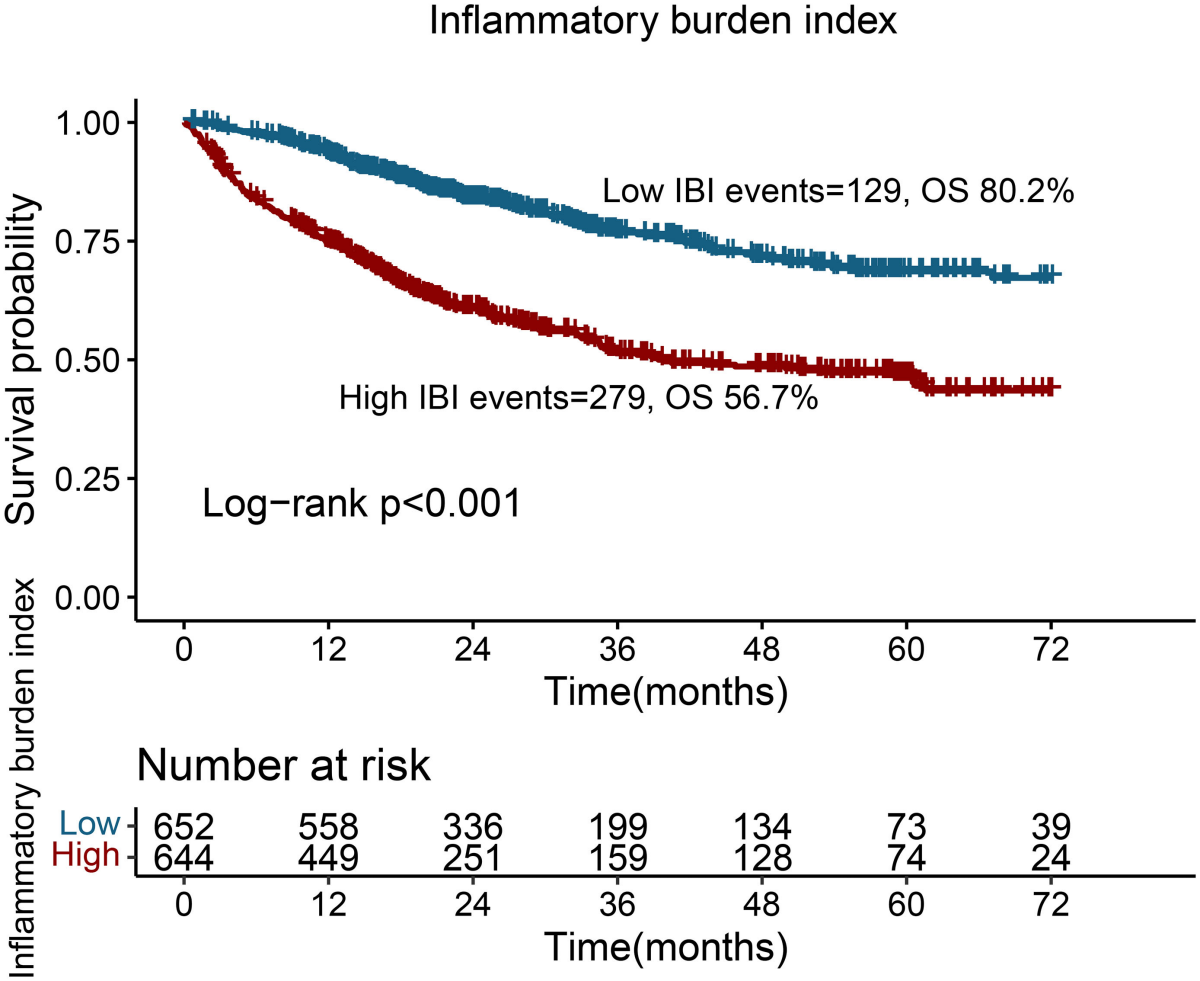
Kaplan-Meier curve of inflammatory burden index in CRC patients.

Patients with a high IBI had significantly worse OS than those with a low IBI (56.7% vs. 80.2%; log-rank p<0.001) (**Figure 3**). For stage III disease, patients with high IBI had a worse prognosis than those with low IBI (72.7% vs. 90.1%; log-rank p < 0.001). For stage IV, IBI was able to effectively differentiate the prognosis of CRC patients (31.3% vs. 57.1%; log-rank p<0.001). However, for early-stage tumors, IBI failed to produce a statistically significant difference in the prognosis assessment (**Figure S3**). We further conducted a subgroup analysis based on different anticancer therapies and found that IBI could effectively differentiate the prognosis of the population receiving radiotherapy, chemotherapy, or surgery (**Figure S4**).

### Relationship between IBI and survival of CRC patients

Univariate and multivariate-adjusted restricted cubic spline plots showed an inverted L-shaped dose-response relationship between continuous IBI and the survival of CRC patients; that is, with an increase in continuous IBI, the prognosis of CRC patients gradually worsened (**Figure S5**). Multivariate Cox regression analysis showed that continuous IBI was an independent risk factor for the prognosis of CRC patients (HR = 1.165, 95% CI = 1.043–1.302, p < 0.001). Dichotomic IBI was also an independent factor affecting CRC patients (HR = 2.431, 95% CI = 1.951–3.028, P < 0.001). Compared with the Q1 group, patients in the Q2, Q3, and Q4 groups had a progressively higher risk of poor prognosis, with HRs of 1.248, 2.120, and 3.519, respectively (**Table 1**). We further excluded confounding diseases for the sensitivity analyses, including Crohn’s disease and ulcerative colitis. The results showed that IBI was still an independent factor affecting the prognosis of CRC patients (HR = 1.174, 95% CI = 1.052–1.311, p = 0.004) (**Table S8**). We then conducted a multivariate-adjusted subgroup analysis, and the results showed that high IBI was an independent risk factor affecting most of the subgroups (**Figure S6**).

**Table 1:** Association between inflammatory burden index and overall survival of patients with colorectal cancer.

IBI	Model a	p value	Model b	p value	Model c	p value
IBI	1.242 (1.123,1.374)	<0.001	1.161 (1.044,1.291)	0.006	1.165 (1.043,1.302)	0.007
Cutoff value		<0.001		<0.001		<0.001
C1 (<16)	ref		ref		ref	
C2 (≥16)	2.629 (2.133,3.239)		2.541 (2.057,3.14)		2.431 (1.951,3.028)	
Quartiles						
Q1 (<4.08)	ref		ref		ref	
Q2 (4.08-11.37)	1.522 (1.074,2.155)	0.018	1.287 (0.907,1.826)	0.158	1.248 (0.878,1.774)	0.218
Q3 (11.37-65.47)	2.643 (1.927,3.626)	<0.001	2.23 (1.618,3.074)	<0.001	2.120 (1.527,2.941)	<0.001
Q4 (≥65.47)	3.795 (2.804,5.136)	<0.001	3.658 (2.692,4.971)	<0.001	3.519 (2.559,4.839)	<0.001
p for trend		<0.001		<0.001		<0.001

Model a: No adjusted.

Model b: Adjusted for age, sex, BMI, TNM stage.

Model c: Adjusted for age, sex, BMI, TNM stage, surgery, radiotherapy, chemotherapy, hypertension, diabetes, smoking, drinking, family history.

### Relationship between IBI and secondary outcomes

Using short-term outcomes as the dependent variable, we explored the impact of IBI changes on it. Multivariate logistic regression analysis showed that IBI was an independent factor affecting short-term outcomes (OR = 1.537, 95% CI = 1.258–1.878, p < 0.001). Patients with high IBI had a more than the 4-fold higher risk of adverse short-term outcomes than those with low IBI (OR = 5.816, 95% CI = 2.686–12.596, p < 0.001) (**Table S9**). Interestingly, IBI was found to be an independent factor affecting malnutrition (OR = 2.996, 95% CI = 1.471–6.103, p = 0.003). With an increase in IBI, the risk of malnutrition gradually increases. Compared to the Q1 group, the ORs of the Q2, Q3, and Q4 groups were 1.163, 2.241, and 5.017, respectively. Dichotomous IBI was also independently associated with recurrence in CRC patients (OR = 1.744, 95% CI = 1.176–2.587, p = 0.006) (**Table S9**).

### Randomized internal validation

**Figure 4:**
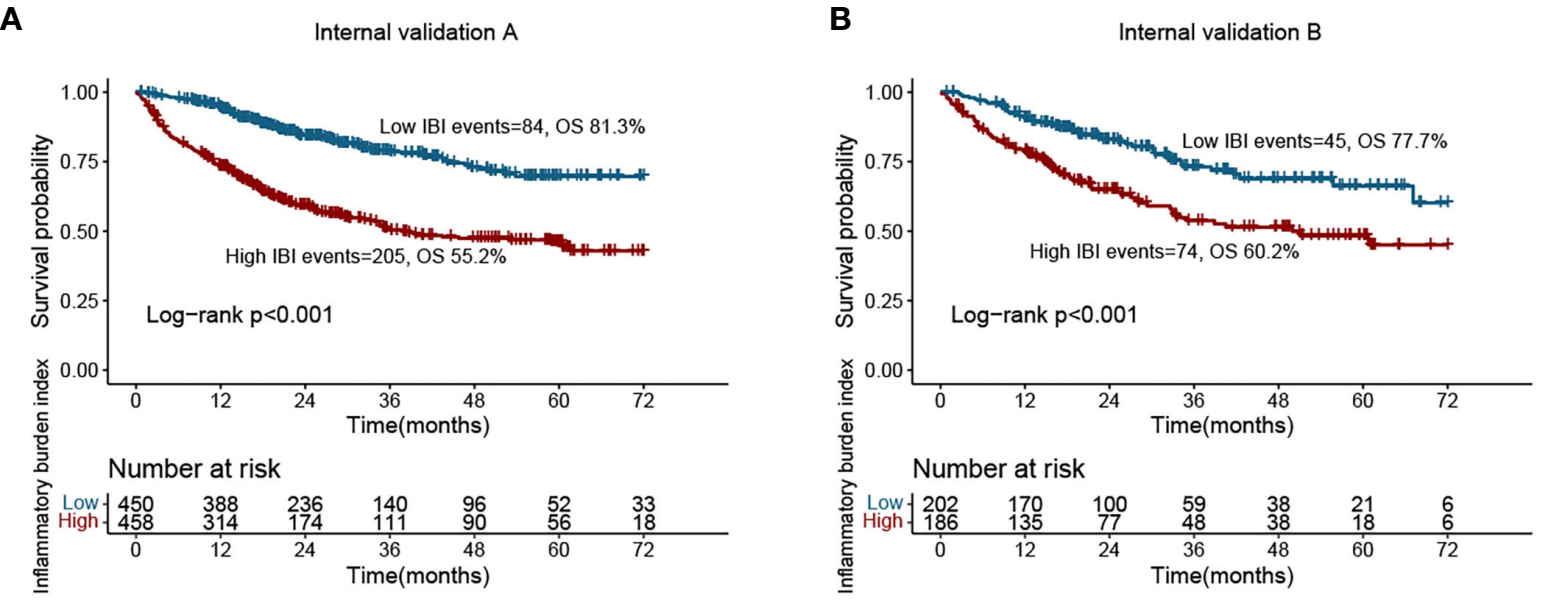
Kaplan-Meier curve of inflammatory burden index in CRC patients at internal validation cohorts. **(A)** Validation cohort A; **(B)** Validation cohort B.

We randomly divided the total population into validation cohorts A (908 cases) and B (388 cases) at a ratio of 7:3 for randomized internal validation. **Table S10** presents the clinicopathological characteristics of the two cohorts. We found that among these systemic inflammation biomarkers, IBI also had optimal prediction accuracy in the validation cohorts (**Table S11**). Subsequent survival curves showed that patients with high IBI had a worse prognosis than those with low IBI in the validation cohorts (validation A, 55.2% vs. 81.3%; validation B, 60.2% vs. 77.7%) (**Figure 4**). In validation cohort A, high IBI was an independent risk factor for the prognosis of CRC patients (HR = 1.176, 95% CI = 1.021–1.354, p = 0.025). Similarly, in validation cohort B, high IBI was independently associated with poor prognosis in CRC patients (HR = 1.285, 95% CI = 1.023–1.612, p = 0.031) (**Table 2**).

**Table 2:** Association between inflammatory burden index and overall survival of patients with cancer at validation cohorts.

Validation cohort A						
IBI	Model a	p value	Model b	p value	Model c	p value
Continuous (per SD)	1.253 (1.103,1.424)	0.001	1.173 (1.026,1.342)	0.02	1.176 (1.021,1.354)	0.025
Cutoff value		<0.001		<0.001		<0.001
C1 (<16)	ref		ref		ref	
C2 (≥16)	2.905 (2.253,3.746)		2.923 (2.257,3.785)		2.807 (2.15,3.664)	
Quartiles						
Q1 (<4.09)	ref		ref		ref	
Q2 (4.09-10.19)	1.868 (1.212,2.88)	0.005	1.435 (0.929,2.218)	0.104	1.409 (0.909,2.184)	0.125
Q3 (10.19-55.36)	3.264 (2.193,4.858)	<0.001	2.702 (1.805,4.043)	<0.001	2.614 (1.733,3.944)	<0.001
Q4 (≥55.36)	4.715 (3.217,6.911)	<0.001	4.574 (3.107,6.735)	<0.001	4.425 (2.969,6.593)	<0.001
p for trend		<0.001		<0.001		<0.001
Validation cohort B						
IBI	Model a	p value	Model b	p value	Model c	p value
Continuous (per SD)	1.312 (1.091,1.577)	0.004	1.258 (1.03,1.536)	0.025	1.285 (1.023,1.612)	0.031
Cutoff value		<0.001		0.001		0.008
C1 (<16)	ref		ref		ref	
C2 (≥16)	2.095 (1.446,3.036)		1.872 (1.284,2.729)		1.73 (1.151,2.601)	
Quartiles						
Q1 (<3.63)	ref		ref		ref	
Q2 (3.63-9.12)	1.212 (0.67,2.194)	0.524	1.237 (0.676,2.262)	0.49	1.182 (0.639,2.186)	0.594
Q3 (9.12-59.95)	1.767 (1.019,3.065)	0.043	1.576 (0.9,2.761)	0.112	1.385 (0.762,2.516)	0.286
Q4 (≥59.95)	2.578 (1.529,4.348)	<0.001	2.456 (1.446,4.171)	0.001	2.234 (1.265,3.948)	0.006
p for trend		<0.001		<0.001		0.003

Model a: No adjusted.

Model b: Adjusted for age, sex, BMI, TNM stage.

Model c: Adjusted for age, sex, BMI, TNM stage, surgery, radiotherapy, chemotherapy, hypertension, diabetes, smoking, drinking, family history.

## Discussion

Systemic inflammation plays a crucial role in cancer progression and is closely associated with cancer patient survival (6, 17). However, few studies have compared the performance of existing systemic inflammation biomarkers in predicting the prognosis of CRC patients. Herein, we systematically and comprehensively compared the value of 15 systemic inflammation biomarkers consisting of peripheral blood characteristics for the prognosis of CRC patients. Among the numerous systemic inflammation biomarkers, we found that IBI has optimal accuracy in predicting the prognosis of CRC patients. Moreover, a high IBI was an independent risk factor for short-term outcomes, malnutrition, and recurrence in CRC patients. Our study is the first to report and validate IBI as a potential biomarker for predicting prognosis in CRC patients and outperforms previously published and other novel systemic inflammation biomarkers. We summarized the performance of existing systemic inflammation biomarkers in the prognostic assessment of CRC patients and rated them as biomarkers with high, moderate, and low predictive accuracy, providing a valuable reference for the clinical selection of these systemic inflammation biomarkers. We suggest that systemic inflammation biomarkers with high predictive accuracy, including IBI, NC, CAR, and LCR, should be the first choice for prognostic assessment in CRC patients.

Subsequently, we focused on exploring the value of IBI in the prognostic assessment of CRC patients. We found that an elevated IBI was significantly associated with established clinicopathological factors for disease development, including aging, low nutritional status, high inflammatory status, and advanced pathological stage. Elevated IBI was significantly associated with poor prognosis and was an independent prognostic factor affecting all-cause mortality in CRC patients. The randomized internal validation cohort further confirmed the importance of IBI in the prognostic assessment of CRC patients. As a newly developed prognostic marker, the optimal cut-off value for IBI in the CRC population is uncertain. Here we determined the optimal threshold for IBI as 10 in CRC patients. Based on this threshold, IBI can significantly predict the unfavorable prognosis of CRC patients. Patients with high IBI were more than twice as likely to have a worse prognosis than those with low IBI.

Currently, the pathological stage is the most effective tool to help clinicians evaluate prognosis, select treatment modalities, and formulate follow-up strategies for CRC patients (18). However, even CRC patients with the same pathologic stage can have different survival outcomes (19). Cancer-related inflammation can be an important reason for these differences. Here, we found that IBI was effective in differentiating outcomes among patients with advanced disease (stage III and IV); however, the difference was not statistically significant among patients with early-stage disease (stage I–II). In addition, high IBI was also an independent risk factor for recurrence in CRC patients. We hypothesized that this might be due to the inflammatory burden being in a correctable state in the early stages of the disease. As tumors progress, it becomes increasingly difficult to reverse inflammation and thus plays an important role in the prognostic assessment. This suggests that IBI monitoring can serve as a reference for monitoring disease progression and efficacy. In addition, we found that the combination of IBI (host status) and TNM (tumor status) may increase the added benefit of prognostic prediction. As a simple, easy to obtain and relatively noninvasive prognostic biomarker compared to invasive pathological procedures, IBI may provide additional prognostic value for CRC patients in addition to the pathological stage.

CRP is the most representative biomarker of systemic inflammation and is widely used in routine clinical practice (20, 21). Elevated circulating CRP levels are significantly associated with poor outcomes and increased mortality in CRC patients (22, 23). Neutrophils and lymphocytes are the first line of defense against cancer. Neutrophils are activated and play a role in chemotaxis, phagocytosis, intracellular killing, and regulation of adaptive immunity under high inflammatory conditions (24). Lymphocytes play an important role in tumor immune surveillance by inducing cytotoxic cell death and inhibiting the proliferation and growth of tumor cells (25). Neutrophil/lymphocyte-based biomarkers have been widely reported to be associated with poor outcomes of cancer (17, 26). Combining the strengths of its constituent parameters, IBI comprehensively reflects the inflammatory and immune status of the body and is a promising prognostic biomarker. The findings of our study may provide insights into the nature of the relationship between systemic inflammation and the survival of CRC patients, thus providing a valuable reference for the selection of systemic inflammation biomarkers for prognosis assessment, efficacy prediction, and follow-up monitoring of CRC patients.

Our study has some limitations. First, we found that IBI-led systemic inflammation biomarkers with high predictive accuracy had optimal predictive accuracy in CRC and were validated by internal cohorts. However, these results require external validation in other cohorts. Since this study was based on clinical routine blood biochemical parameters and data of other molecular plasma-based biomarkers (e.g., circulating cell-free DNA) were lacking, we were unable to further investigate their prognostic efficacy and combined effects in CRC. Second, owing to the lack of ongoing surveillance data, we were unable to observe the impact of the trajectory of these systemic inflammation biomarkers on the prognosis of CRC. Finally, this study may have been affected by differences in analytical instrumentation and tumor treatment among different cohorts. Larger and more rigorous prospective trials are required to overcome these obstacles.

## Conclusion

As a reflection of systemic inflammation, IBI is a feasible and promising biomarker to assess the prognosis of CRC patients. Compared with other systemic inflammation biomarkers, IBI showed optimal predictive accuracy and is recommended for routine use in CRC patients.

## Data availability statement

The raw data supporting the conclusions of this article will be made available by the authors, without undue reservation.

## Ethics statement

The studies involving human participants were reviewed and approved by Beijing Shijitan Hospital, Capital Medical University. The patients/participants provided their written informed consent to participate in this study.

## Author contributions

HS had full access to all the data in the study and take responsibility for the integrity of the data and the accuracy of the data analysis. Study concept and design, HX, GR, and LW. Acquisition, analysis, or interpretation of data, HX, GR, and LW. Drafting of the manuscript, HX. Critical revision of the manuscript for important intellectual content, all authors. Obtained funding, HS. Administrative, technical, or material support, HS. Supervision, HX, HS, and LD. Final approval of manuscript, all authors. All authors contributed to the article and approved the submitted version.
