# Supplementary material for: Comprehensive comparative analysis of prognostic value of serum systemic inflammation biomarkers for colorectal cancer: Results from a large multicenter collaboration
Source: Front Immunol. 2023 Jan 5;13:1092498. doi: 10.3389/fimmu.2022.1092498 (PMC9849562; doi:10.3389/fimmu.2022.1092498)
Supplement: Supplementary file 1 [file DataSheet_1.docx]

**Figure S1.** Cut-off of IBI in patients with colorectal cancer.

**
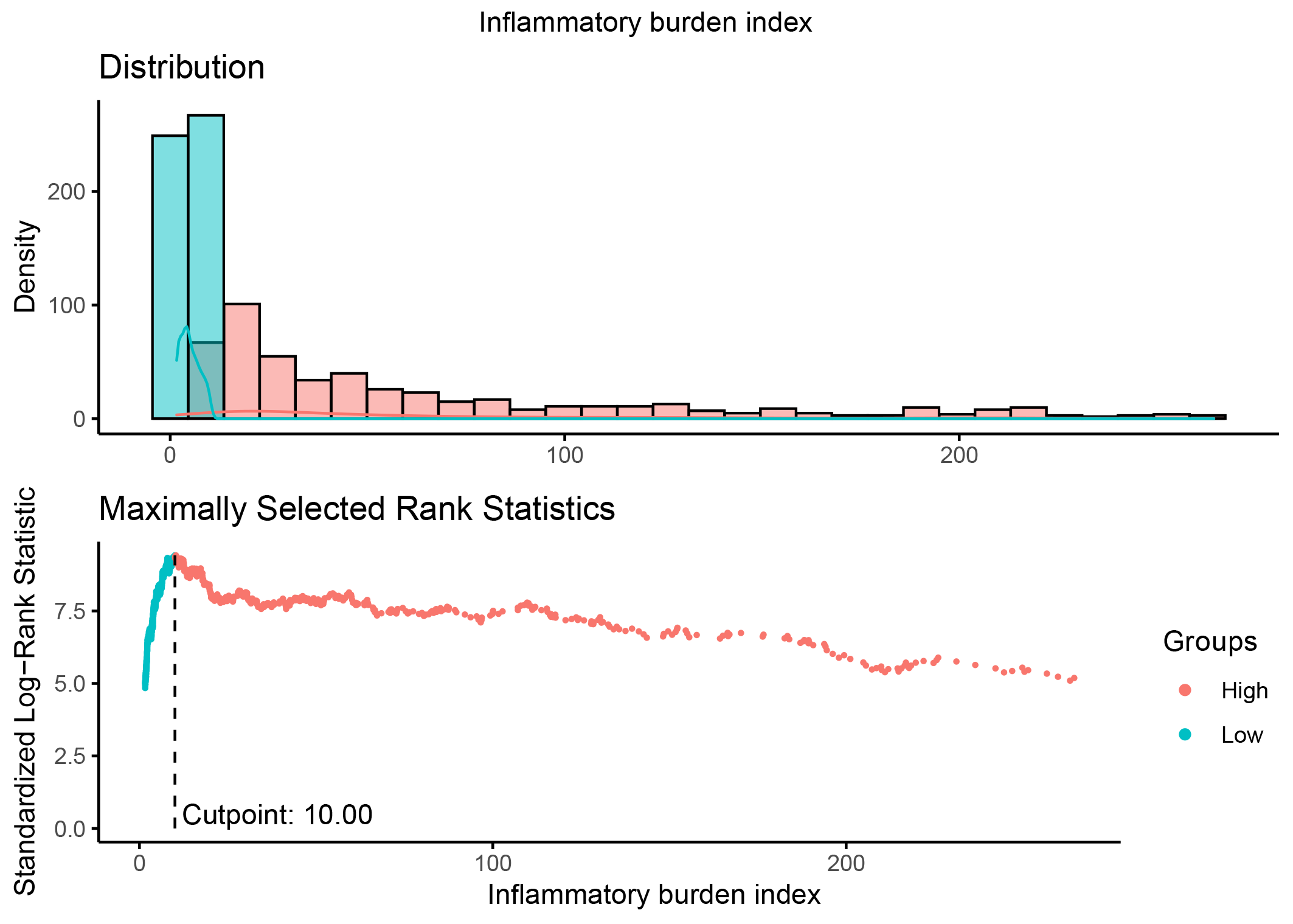
**

**Figure S2.** Spearman's rank correlations between systemic inflammatory and patient characteristics.


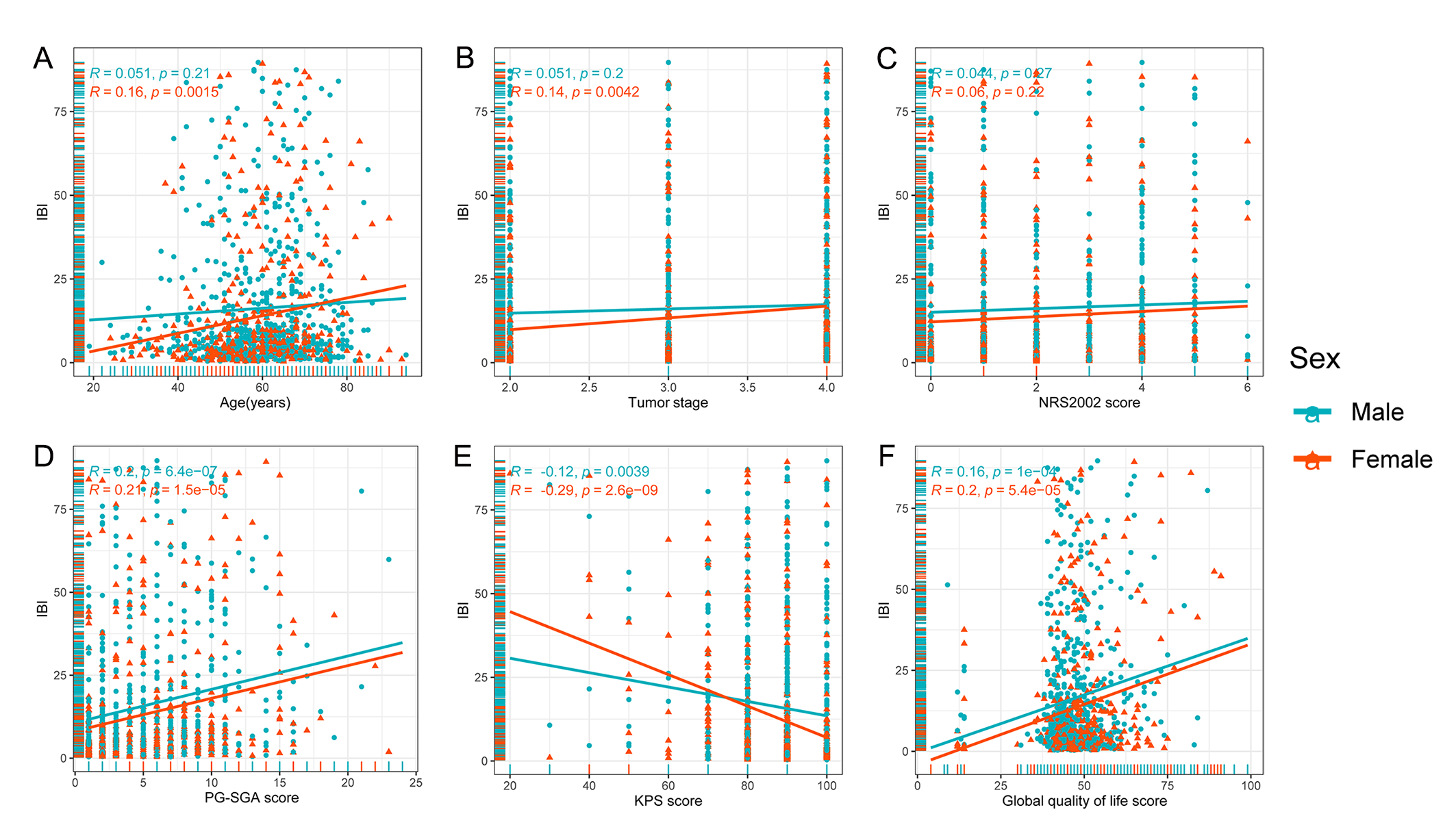


Notes: (A) IBI vs. Age. (B) IBI vs. Tumor stage. (C) IBI vs. NRS2002 score. (D) IBI vs. PG-SGA score. (E) IBI vs. KPS score. (F) IBI vs. Global quality of life score.

**Figure S3.** Subgroup survival analysis of inflammatory burden index based on pathological stage.


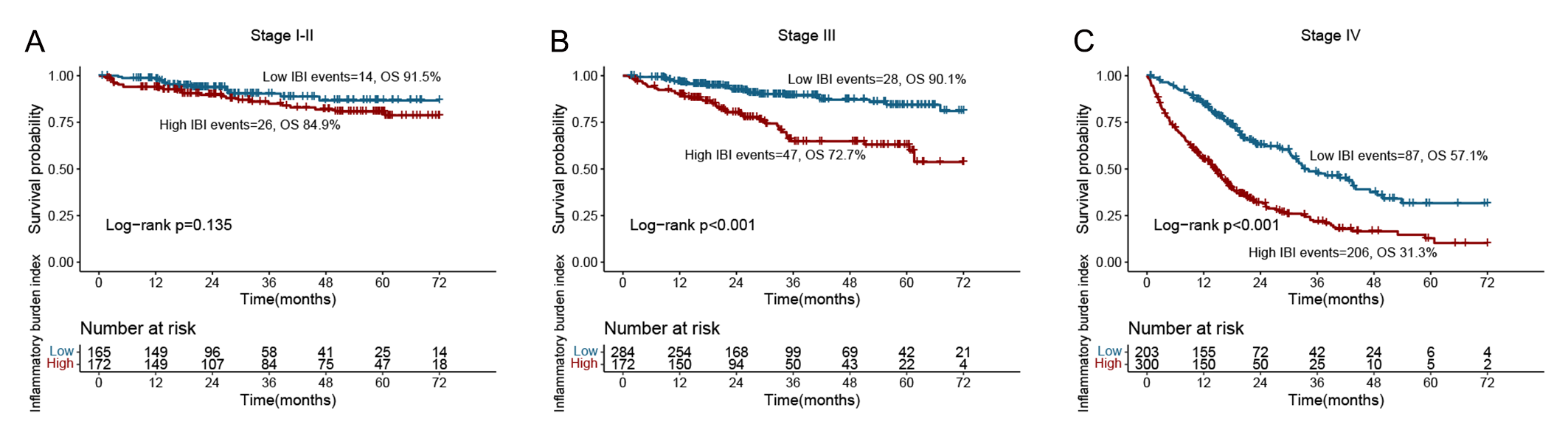


**Notes：**A, Stage I-II; B, Stage III; C, Stage IV.

**Figure S4.** Subgroup survival analysis of inflammatory burden index based on anti-tumor therapy method.

**
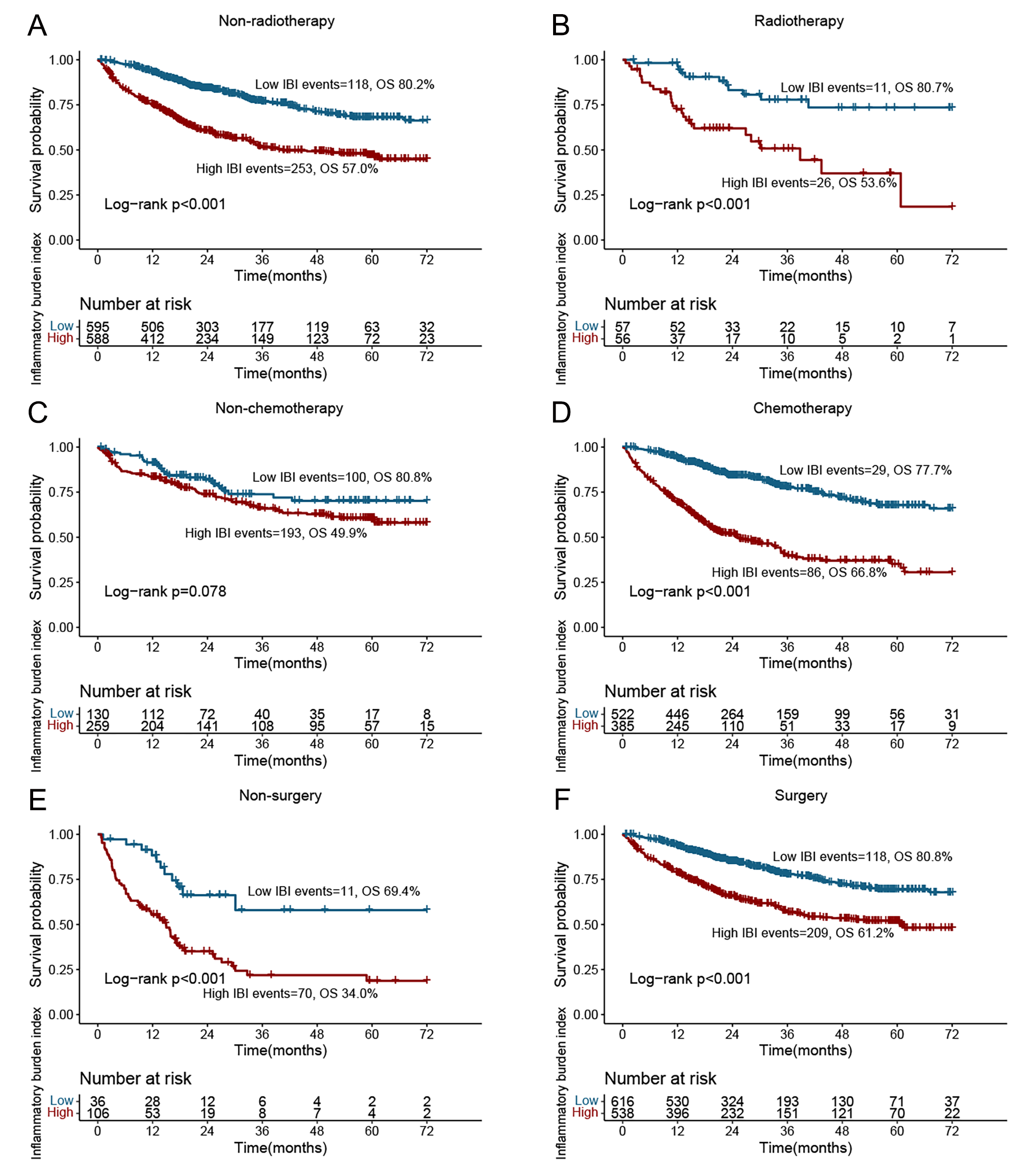
**

**Notes：**A, Non-radiotherapy; B, Radiotherapy; C, Non-chemotherapy; D, Chemotherapy; E, Non-surgery; F, Surgery.

**Figure S5.** The association between inflammatory burden index and survival in CRC patients.
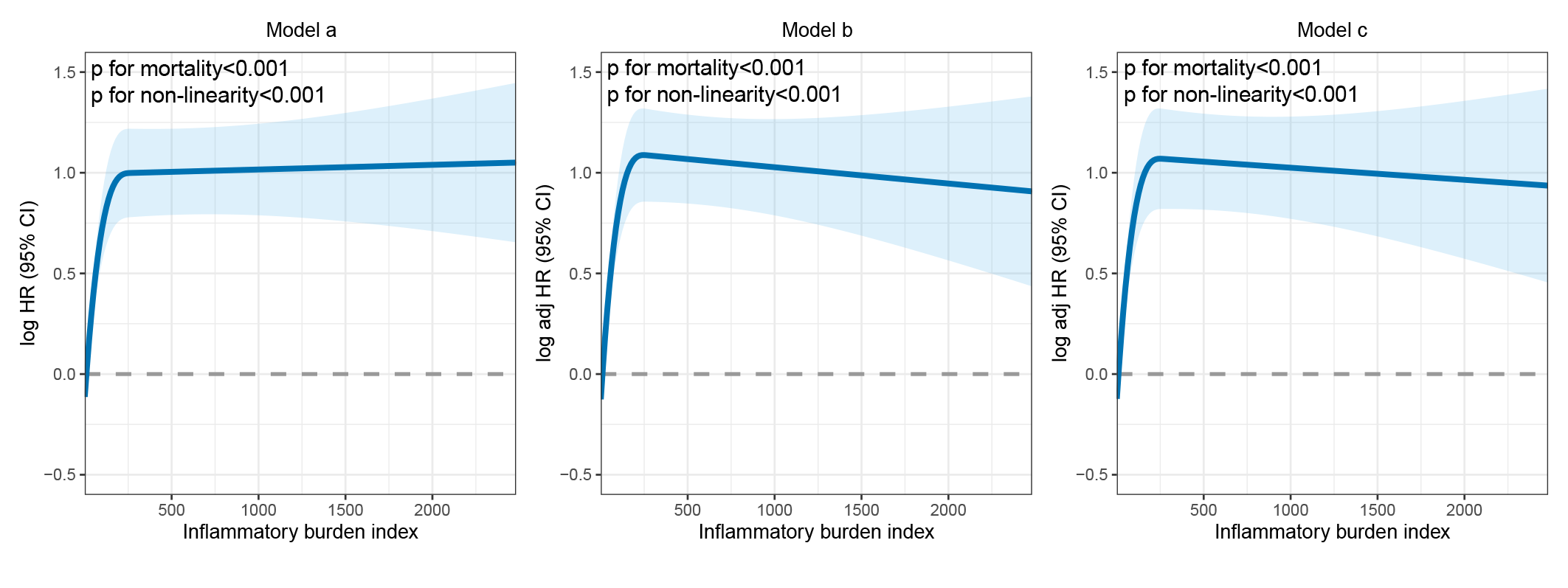


Notes:

Model a: No adjusted.

Model b: Adjusted for age, sex, BMI, TNM stage.

Model c: Adjusted for age, sex, BMI, TNM stage, surgery, radiotherapy, chemotherapy, hypertension, diabetes, smoking, drinking, family history.

**Figure S6.** The association between inflammatory burden index and hazard risk of overall survival in various subgroups.





**Notes:** The model adjusted for age, sex, BMI, TNM stage, surgery, radiotherapy, chemotherapy, hypertension, diabetes, smoking, drinking, family history.

**Table S1.** The sample size of INSCOC was calculated using the following formula:

1. Sample size was estimated based on simple random sampling (SRS). The rate of malnutrition among cancer inpatients in China was about 26.58%, which was used as an estimate of π in this study;α=0.05, Zα/2=1.96; The absolute maximum allowable error δ (about 10% of the incidence) = p-π.
2. The calculation formula is: N (SRS)= Zα/2×π×(1-π)/ δ2; 1,052 people per floor;
3. The DEFF of design effect is 2.5, 2,630 people need to be investigated in per floor;
4. According to the east, south, west, north, and central regions of China and gender, the calculated total sample size was 2630×5 (east, south, west, north, and central) ×2 (gender) =26,300.

In this study, we enrolled 1296 CRC patients from the INSCOC database.

**Table S2.** Fifteen inflammatory biomarkers evaluated in this study.

| Biomarker name | Biomarker formulas |
| --- | --- |
| C-reactive protein-to-albumin ratio (CAR) | C-reactive protein (mg/dL)/albumin (g/dL) |
| Platelet-to-albumin ratio (PAR) | Platelet (/uL)/albumin (g/dL) |
| Neutrophil-to-albumin ratio (NAR) | Neutrophil (/uL)/albumin (g/dL) |
| Lymphocyte-to-C-reactive protein ratio (LCR) | Lymphocyte (/uL)/C-reactive protein (mg/L) |
| Platelet-to-Lymphocyte ratio (PLR) | Platelet (/uL)/lymphocyte (/uL) |
| Neutrophil-to-Lymphocyte ratio (NLR) | Neutrophil (/uL)/lymphocyte (/uL) |
| Systemic-Immune-Inflammation Index (SII) | Platelet (/uL)×Neutrophil (/uL)/lymphocyte (/uL) |
| C-reactive protein-Neutrophil-Lymphocyte ratio (Inflammatory burden index, IBI) | C-reactive protein (mg/L)×Neutrophil (/uL)/lymphocyte (/uL) |
| Glasgow Prognostic Score (GPS) | C-reactive protein≤10mg/L and albumin ≥35g/L: 0 score  C-reactive protein≤10mg/L or albumin < 35g/L: 1 score  C-reactive protein >10mg/L and albumin < 35g/L: 2 score |
| Modified Glasgow Prognostic Score (mGPS) | C-reactive protein≤10mg/L and albumin ≥35g/L: 0 score  C-reactive protein≤10mg/L and albumin < 35g/L: 0 score  C-reactive protein > 10mg/L: 1 score  C-reactive protein >10mg/L and albumin < 35g/L: 2 score |
| Lymphocyte C-reactive protein score (LCS) | Lymphocyte ≥1×10^9/L and C-reactive protein ≤ 3 mg/L: 0 score;  Lymphocyte ≤1×10^9/L or C-reactive protein ≥ 3 mg/L: 1 score;  Lymphocyte ≤1×10^9/L and C-reactive protein ≥ 3 mg/L: 2 score |
| Neutrophil-C-reactive protein score(NC) | Lymphocyte (/uL) × C-reactive protein score (mg/L) |
| Platelet-C-reactive protein score (PC) | Platelet (/uL) × C-reactive protein score (mg/L) |
| Neutrophil-Platelet score (PC) | Neutrophil (/uL) × Platelet score (/uL) |
| Lymphocyte-Albumin score (LA) | Lymphocyte (/uL) × Albumin (g/dL) score |

**Table S3.** The clinicopathological features in CRC patients.

| **Characteristic** | Overall  n=1296 |
| --- | --- |
|  |  |
| Sex, male, n (%) | 787 ( 60.7) |
| Age, years, mean (SD) | 59.62 (11.77) |
| BMI (median (IQR)) | 22.58 (20.54, 24.84) |
| Hypertension, yes, n (%) | 286 ( 22.1) |
| Diabetes, yes, n (%) | 142 ( 11.0) |
| Smoking yes, n (%) | 521 ( 40.2) |
| Drinking, yes, n (%) | 278 ( 21.5) |
| Family history, yes, n (%) | 202 ( 15.6) |
| TNM stage, n (%) |  |
| Stage I-II | 337 ( 26.0) |
| Stage III | 456 ( 35.2) |
| Stage IV | 503 ( 38.8) |
| Surgery, yes, n (%) | 1154 ( 89.0) |
| Radiotherapy, yes, n (%) | 113 ( 8.7) |
| Chemotherapy, yes, n (%) | 907 ( 70.0) |
| White blood cells (median (IQR)) | 5.76 (4.42, 7.28) |
| Neutrophil (mean (SD)) | 3.58 (2.50, 5.00) |
| Lymphocyte (mean (SD)) | 1.44 (1.06, 1.84) |
| Platelets (median (IQR)) | 212.00 (164.00, 271.25) |
| Red blood cells (median (IQR)) | 4.24 (3.83, 4.66) |
| Hemoglobin (median (IQR)) | 125.00 (108.00, 138.00) |
| Albumin (mean (SD)) | 39.70 (35.70, 42.70) |
| CRP (median (IQR)) | 3.72 (2.67, 18.02) |
| KPS score (median (IQR)) | 90.00 (80.00, 90.00) |
| PGSGA score (median (IQR)) | 6.00 (2.00, 9.00) |
| Cachexia, yes, n (%) | 495 ( 38.2) |
| Global quality of life score (median (IQR)) | 47.00 (43.00, 52.00) |
| Short-term outcome, yes, n (%) | 67 (5.2) |
| Status, death, n (%) | 408 ( 31.5) |
| LOS (median (IQR)) | 9.00 (5.00, 15.00) |
| Hospitalization expenses(median (IQR)) | 17732.55 (9659.30, 41713.88) |

**Table S4.** The prognostic value of inflammation-related markers in the prognosis assessment.

| Inflammation-related markers | Model a | p value | Model b | p value | Model c | p value |
| --- | --- | --- | --- | --- | --- | --- |
| IBI | 1.242 (1.123,1.374) | <0.001 | 1.161 (1.044,1.291) | 0.006 | 1.165 (1.043,1.302) | 0.007 |
| NC | 1.255 (1.169,1.347) | <0.001 | 1.178 (1.098,1.263) | <0.001 | 1.172 (1.088,1.263) | <0.001 |
| CAR | 1.317 (1.223,1.419) | <0.001 | 1.283 (1.195,1.377) | <0.001 | 1.28 (1.185,1.382) | <0.001 |
| LCR | 0.622 (0.507,0.764) | <0.001 | 0.68 (0.557,0.831) | <0.001 | 0.701 (0.575,0.856) | <0.001 |
| PC | 1.299 (1.211,1.393) | <0.001 | 1.229 (1.145,1.32) | <0.001 | 1.201 (1.112,1.297) | <0.001 |
| NAR | 1.365 (1.269,1.469) | <0.001 | 1.394 (1.297,1.498) | <0.001 | 1.368 (1.267,1.478) | <0.001 |
| mGPS |  | <0.001 |  | <0.001 |  | <0.001 |
| 0 score | ref |  | ref |  | ref |  |
| 1 score | 1.908 (1.498,2.431) | <0.001 | 1.738 (1.363,2.216) | <0.001 | 1.67 (1.302,2.142) | <0.001 |
| 2 score | 2.74 (2.161,3.473) | <0.001 | 3.019 (2.37,3.847) | <0.001 | 2.873 (2.225,3.71) | <0.001 |
| NLR | 1.137 (0.972,1.331) | 0.108 | 1.18 (0.998,1.395) | 0.053 | 1.16 (0.971,1.385) | 0.103 |
| SII | 1.229 (1.138,1.328) | <0.001 | 1.256 (1.157,1.364) | <0.001 | 1.241 (1.138,1.354) | <0.001 |
| NP | 1.315 (1.223,1.414) | <0.001 | 1.312 (1.223,1.407) | <0.001 | 1.285 (1.192,1.384) | <0.001 |
| LCS |  | <0.001 |  | <0.001 |  | <0.001 |
| 0 score | ref |  | ref |  | ref |  |
| 1 score | 2.618 (1.902,3.603) | <0.001 | 2.197 (1.594,3.028) | <0.001 | 2.04 (1.475,2.823) | <0.001 |
| 2 score | 3.965 (2.764,5.689) | <0.001 | 2.919 (2.026,4.205) | <0.001 | 2.806 (1.938,4.063) | <0.001 |
| GPS |  | <0.001 |  | <0.001 |  | <0.001 |
| 0 score | ref |  | ref |  | ref |  |
| 1 score | 1.764 (1.404,2.217) | <0.001 | 1.504 (1.193,1.896) | 0.001 | 1.413 (1.113,1.795) | 0.005 |
| 2 score | 2.828 (2.218,3.606) | <0.001 | 3.051 (2.378,3.916) | <0.001 | 2.852 (2.191,3.714) | <0.001 |
| PAR | 1.355 (1.246,1.474) | <0.001 | 1.323 (1.221,1.433) | <0.001 | 1.303 (1.194,1.421) | <0.001 |
| LA | 0.838 (0.757,0.928) | 0.001 | 0.914 (0.825,1.013) | 0.087 | 0.929 (0.835,1.034) | 0.178 |
| PLR | 1.09 (0.965,1.231 | 0.166 | 1.11 (0.982,1.256) | 0.096 | 1.092 (0.961,1.241) | 0.177 |

Notes:

Model a: No adjusted.

Model b: Adjusted for age, sex, BMI, TNM stage.

Model c: Adjusted for age, sex, BMI, TNM stage, surgery, radiotherapy, chemotherapy, hypertension, diabetes, smoking, drinking, family history.

**Table S5.** Comparative analysis of the discrimination of each inflammation-related markers for all-cause mortality in CRC.

| Discrimination Ability | C-statistic | Difference | p value |
| --- | --- | --- | --- |
| IBI | 0.667(0.639,0.694) | Ref |  |
| NC | 0.663(0.635,0.691) | -0.004(-0.013, 0.005) | 0.410 |
| CAR | 0.661(0.633,0.689) | -0.006(-0.016, 0.003) | 0.244 |
| LCR | 0.658(0.631,0.686) | -0.009(-0.019, 0.001) | 0.094 |
| PC | 0.651(0.623,0.68) | -0.015(-0.028, -0.003) | 0.013 |
| NAR | 0.634(0.605,0.663) | -0.033(-0.056, -0.007) | 0.010 |
| GPS | 0.624(0.597,0.651) | -0.043(-0.062, -0.023) | <0.001 |
| mGPS | 0.621(0.594,0.647) | -0.046(-0.065, -0.027) | <0.001 |
| NLR | 0.62(0.592,0.649) | -0.046(-0.068, -0.024) | <0.001 |
| SII | 0.616(0.587,0.645) | -0.051(-0.073, -0.027) | <0.001 |
| NP | 0.608(0.578,0.637) | -0.059(-0.085, -0.031) | <0.001 |
| LCS | 0.603(0.58,0.626) | -0.064(-0.089, -0.041) | <0.001 |
| PAR | 0.577(0.547,0.607) | -0.090(-0.119, -0.059) | <0.001 |
| LA | 0.568(0.537,0.598) | -0.099(-0.134, -0.065) | <0.001 |
| PLR | 0.560(0.529,0.591) | -0.107(-0.176, -0.007) | 0.013 |

Table note: cNRI, continuous net reclassification improvement; IDI, integrated discrimination improvement; AGR, Albumin-Globulin ratio; KPS, Karnofsky Performance Status; MAC, mid-arm circumference; HGS, hand grip strength; PG-SGA, patient-generated subjective nutrition assessment.

**Table S6.** Comparative analysis of the discrimination of each inflammation-related markers for all-cause mortality in CRC.

| Discrimination Ability | C-statistic | Difference | p value |
| --- | --- | --- | --- |
| TNM stage | 0.729(0.706,0.751) | Ref |  |
| TNM stage+IBI | 0.782(0.759,0.805) | 0.053(0.042, 0.064) | <0.001 |
| TNM stage+NC | 0.783(0.760,0.807) | 0.054(0.044, 0.067) | <0.001 |
| TNM stage+CAR | 0.781(0.758,0.805) | 0.053(0.041, 0.065) | <0.001 |
| TNM stage+LCR | 0.776(0.752,0.800) | 0.047(0.038, 0.056) | <0.001 |
| TNM stage+PC | 0.777(0.754,0.800) | 0.049(0.038, 0.060) | <0.001 |
| TNM stage+NAR | 0.777(0.754,0.801) | 0.049(0.037, 0.062) | <0.001 |
| TNM stage+GPS | 0.770(0.746,0.794) | 0.041(0.031, 0.052) | <0.001 |
| TNM stage+mGPS | 0.770(0.746,0.793) | 0.041(0.031, 0.051) | <0.001 |
| TNM stage+NLR | 0.768(0.744,0.792) | 0.039(0.031, 0.049) | <0.001 |
| TNM stage+SII | 0.766(0.742,0.789) | 0.037(0.027, 0.048) | <0.001 |
| TNM stage+NP | 0.765(0.742,0.788) | 0.036(0.025, 0.048) | <0.001 |
| TNM stage+LCS | 0.756(0.733,0.779) | 0.028(0.019, 0.035) | <0.001 |
| TNM stage+PAR | 0.754(0.731,0.777) | 0.025(0.013, 0.038) | <0.001 |
| TNM stage+LA | 0.748(0.724,0.773) | 0.020(0.010, 0.029) | <0.001 |
| TNM stage+PLR | 0.746(0.722,0.770) | 0.017(0.001, 0.039) | 0.071 |

Table note: cNRI, continuous net reclassification improvement; IDI, integrated discrimination improvement; AGR, Albumin-Globulin ratio; KPS, Karnofsky Performance Status; MAC, mid-arm circumference; HGS, hand grip strength; PG-SGA, patient-generated subjective nutrition assessment.

**Table S7.** Characteristics by level of inflammatory burden index in CRC patients.

| **Characteristic** | IBI | | |
| --- | --- | --- | --- |
|  | Low n=652 | High n=644 | p value |
| Sex, male, n (%) | 369 ( 56.6) | 418 ( 64.9) | 0.003 |
| Age, years, mean (SD) | 58.22 (11.60) | 61.04 (11.77) | <0.001 |
| BMI (median (IQR)) | 22.68 (20.66, 24.90) | 22.48 (20.37, 24.80) | 0.122 |
| Hypertension, yes, n (%) | 125 ( 19.2) | 161 ( 25.0) | 0.014 |
| Diabetes, yes, n (%) | 54 ( 8.3) | 88 ( 13.7) | 0.003 |
| Smoking yes, n (%) | 237 ( 36.3) | 284 ( 44.1) | 0.005 |
| Drinking, yes, n (%) | 124 ( 19.0) | 154 ( 23.9) | 0.038 |
| Family history, yes, n (%) | 105 ( 16.1) | 97 ( 15.1) | 0.66 |
| TNM stage, n (%) |  |  | <0.001 |
| Stage I-II | 165 ( 25.3) | 172 ( 26.7) |  |
| Stage III | 284 ( 43.6) | 172 ( 26.7) |  |
| Stage IV | 203 ( 31.1) | 300 ( 46.6) |  |
| Surgery, yes, n (%) | 616 ( 94.5) | 538 ( 83.5) | <0.001 |
| Radiotherapy, yes, n (%) | 57 ( 8.7) | 56 ( 8.7) | 0.999 |
| Chemotherapy, yes, n (%) | 522 ( 80.1) | 385 ( 59.8) | <0.001 |
| White blood cells (median (IQR)) | 4.99 (4.07, 6.13) | 6.80 (5.41, 8.88) | <0.001 |
| Neutrophil (mean (SD)) | 2.80 (2.05, 3.64) | 4.68 (3.44, 6.43) | <0.001 |
| Lymphocyte (mean (SD)) | 1.58 (1.21, 1.96) | 1.30 (0.90, 1.70) | <0.001 |
| Platelets (median (IQR)) | 198.00 (157.75, 246.25) | 231.50 (176.75, 295.25) | <0.001 |
| Red blood cells (median (IQR)) | 4.33 (3.97, 4.72) | 4.12 (3.69, 4.57) | <0.001 |
| Hemoglobin (median (IQR)) | 129.00 (116.00, 141.00) | 120.00 (101.00, 134.00) | <0.001 |
| Albumin (mean (SD)) | 41.30 (38.60, 43.70) | 37.00 (33.30, 40.90) | <0.001 |
| CRP (median (IQR)) | 2.97 (0.90, 3.20) | 18.15 (6.59, 45.78) | <0.001 |
| KPS score (median (IQR)) | 90.00 (80.00, 90.00) | 80.00 (80.00, 90.00) | <0.001 |
| PGSGA score (median (IQR)) | 4.00 (2.00, 7.00) | 7.00 (4.00, 10.00) | <0.001 |
| Cachexia, yes, n (%) | 207 ( 31.7) | 288 ( 44.7) | <0.001 |
| Global quality of life score (median (IQR)) | 45.00 (42.00, 50.00) | 49.00 (44.00, 56.00) | <0.001 |
| Short-term outcome, yes, n (%) | 8 (1.2) | 59 ( 9.2) | <0.001 |
| Status, death, n (%) | 129 (19.8) | 279 ( 43.3) | <0.001 |
| LOS (median (IQR)) | 8.00 (4.00, 12.00) | 11.00 (7.00, 17.00) | <0.001 |
| Hospitalization expenses(median (IQR)) | 13938.65 (8002.73, 25318.65) | 24700.05 (13693.08, 52815.12) | <0.001 |

**Table S8.** The sensitivity analysis of the relationship between inflammatory burden index and survival.

| Excluding patients with Miscellaneous diseases, including Crohn's disease and ulcerative colitis | | | | | | |
| --- | --- | --- | --- | --- | --- | --- |
| IBI* | Model a | p value | Model b | p value | Model c | p value |
| Continuous (per SD) | 1.251 (1.132,1.381) | <0.001 | 1.165 (1.048,1.294) | 0.005 | 1.174 (1.052,1.311) | 0.004 |
| Cutoff value |  | <0.001 |  | <0.001 |  | <0.001 |
| C1 (<16) | ref |  | ref |  | ref |  |
| C2 (≥16） | 2.627 (2.128,3.243) |  | 2.548 (2.059,3.153) |  | 2.441 (1.956,3.047) |  |
| Quartiles |  |  |  |  |  |  |
| Q1 (<3.90) | ref |  | ref |  | ref |  |
| Q2 (3.90-9.83) | 1.481 (1.043,2.102) | 0.028 | 1.24 (0.872,1.764) | 0.231 | 1.196 (0.839,1.704) | 0.322 |
| Q3 (9.83-56.50) | 2.628 (1.915,3.607) | <0.001 | 2.189 (1.587,3.02) | <0.001 | 2.071 (1.491,2.876) | <0.001 |
| Q4 (≥56.50) | 3.693 (2.725,5.003) | <0.001 | 3.59 (2.639,4.883) | <0.001 | 3.459 (2.512,4.763) | <0.001 |
| p for trend |  | <0.001 |  | <0.001 |  | <0.001 |

Notes:

Model a: No adjusted.

Model b: Adjusted for age, sex, BMI, TNM stage.

Model c: Adjusted for age, sex, BMI, TNM stage, surgery, radiotherapy, chemotherapy, hypertension, diabetes, smoking, drinking, family history.

**Table S9.** Logistic regression analysis of inflammatory burden index associated with secondary outcome.

| **Short-term outcomes (90-day outcomes)** | | | | | | |
| --- | --- | --- | --- | --- | --- | --- |
| IBI | Model a | p value | Model b | p value | Model c | p value |
| Continuous (per SD) | 1.542 (1.272,1.869) | <0.001 | 1.494 (1.224,1.825) | <0.001 | 1.537 (1.258,1.878) | <0.001 |
| Cutoff value |  | <0.001 |  | <0.001 |  | <0.001 |
| C1 (<16) | ref |  | ref |  | ref |  |
| C2 (≥16） | 8.119 (3.847,17.134) |  | 6.279 (2.941,13.406) |  | 5.816 (2.686,12.596) | <0.001 |
| Quartiles |  |  |  |  |  |  |
| Q1 (<4.08) | ref |  | ref |  | ref |  |
| Q2 (4.08-11.37) | 1.677 (0.397,7.077) | 0.482 | 1.364 (0.320,5.815) | 0.675 | 1.356 (0.317,5.805) | 0.682 |
| Q3 (11.37-65.47) | 5.925 (1.719,20.419) | 0.005 | 4.183 (1.196,14.633) | 0.025 | 4.049 (1.144,14.325) | 0.030 |
| Q4 (≥65.47) | 15.936 (4.886,51.974) | <0.001 | 5.250 (3.612,7.631) | <0.001 | 10.256 (3.043,34.563) | <0.001 |
| p for trend |  | <0.001 |  | <0.001 |  | <0.001 |
| **Malnutrition (PGSGA≥4)** | | | | | | |
| IBI | Model a | p value | Model b | p value | Model c | p value |
| Continuous (per SD) | 3.656 (1.769,7.555) | <0.001 | 3.273 (1.594,6.723) | 0.001 | 2.996 (1.471,6.103) | 0.003 |
| Cutoff value |  | <0.001 |  | <0.001 |  | <0.001 |
| C1 (<16) | ref |  | ref |  | ref |  |
| C2 (≥16） | 3.174 (2.49,4.046) |  | 3.097 (2.407,3.985) |  | 2.983 (2.289,3.888) |  |
| Quartiles |  |  |  |  |  |  |
| Q1 (<4.08) | ref |  | ref |  | ref |  |
| Q2 (4.08-11.37) | 1.118 (0.821,1.523) | 0.478 | 1.164 (0.843,1.608) | 0.357 | 1.163 (0.839,1.612) | 0.365 |
| Q3 (11.37-65.47) | 2.211 (1.6,3.056) | <0.001 | 2.260 (1.610,3.174) | <0.001 | 2.241 (1.581,3.179) | <0.001 |
| Q4 (≥65.47) | 5.405 (3.703,7.891) | <0.001 | 5.248 (3.545,7.771) | <0.001 | 5.017 (3.336,7.546) | <0.001 |
| p for trend |  | <0.001 |  | <0.001 |  | <0.001 |
| **Recurrence** | | | | | | |
| IBI | Model a | p value | Model b | p value | Model c | p value |
| Continuous (per SD) | 1.000 (0.834,1.197) | 0.988 | 1.000 (0.999,1.001) | 0.275 | 1.000 (0.999,1.001) | 0.420 |
| Cutoff value |  | <0.001 |  | 0.013 |  | 0.006 |
| C1 (<16) | ref |  | ref |  | ref |  |
| C2 (≥16） | 2.062 (1.606,2.647) |  | 1.597 (1.102,2.315) |  | 1.744 (1.176,2.587) |  |
| Quartiles |  |  |  |  |  |  |
| Q1 (<4.08) | ref |  | ref |  | ref |  |
| Q2 (4.08-11.37) | 1.704 (1.157,2.51) | 0.007 | 1.272 (0.732,2.212) | 0.393 | 1.180 (0.670,2.080) | 0.566 |
| Q3 (11.37-65.47) | 2.776 (1.913,4.028) | <0.001 | 1.915 (1.108,3.311) | 0.020 | 1.896 (1.073,3.350) | 0.028 |
| Q4 (≥65.47) | 2.628 (1.809,3.819) | <0.001 | 1.668 (0.969,2.873) | 0.065 | 1.819 (1.016,3.257) | 0.044 |
| p for trend |  | <0.001 |  | 0.091 |  | 0.069 |

Notes:

Model a: No adjusted.

Model b: Adjusted for age, sex, BMI, TNM stage.

Model c: Adjusted for age, sex, BMI, TNM stage, surgery, radiotherapy, chemotherapy, hypertension, diabetes, smoking, drinking, family history.

**Table S10.** Demographics and clinicopathologic characteristics of patients with colorectal cancer between validation cohorts A and validation cohorts B.

| **Characteristic** | **Validation** **cohorts A**  **n=908** | **Validation cohorts B**  **n=388** | **p value** |
| --- | --- | --- | --- |
| Sex, male, n (%) | 547 (60.2) | 240 (61.9) | 0.629 |
| Age, years, mean (SD) | 59.73 (11.75) | 59.36 (11.75) | 0.599 |
| BMI (median (IQR)) | 22.58 (20.66, 24.84) | 22.59 (20.30, 24.80) | 0.631 |
| Hypertension, yes, n (%) | 201 (22.1) | 85 (21.9) | 0.986 |
| Diabetes, yes, n (%) | 99 (10.9) | 43 (11.1) | 0.999 |
| Smoking yes, n (%) | 368 (40.5) | 153 (39.4) | 0.759 |
| Drinking, yes, n (%) | 188 (20.7) | 90 (23.2) | 0.354 |
| Family history, yes, n (%) | 139 (15.3) | 63 (16.2) | 0.735 |
| TNM stage, n (%) |  |  | 0.682 |
| Stage I-II | 230 (25.3) | 107 (27.6) |  |
| Stage III | 324 (35.7) | 132 (34.0) |  |
| Stage IV | 354 (39.0) | 149 (38.4) |  |
| Surgery, yes, n (%) | 813 (89.5) | 341 (87.9) | 0.439 |
| Radiotherapy, yes, n (%) | 80 ( 8.8) | 33 ( 8.5) | 0.943 |
| Chemotherapy, yes, n (%) | 642 (70.7) | 265 (68.3) | 0.424 |
| White blood cells (median (IQR)) | 5.78 (4.50, 7.42) | 5.61 (4.40, 7.11) | 0.117 |
| Neutrophil (mean (SD)) | 3.60 (2.50, 5.08) | 3.50 (2.43, 4.81) | 0.232 |
| Lymphocyte (mean (SD)) | 1.44 (1.08, 1.84) | 1.44 (1.01, 1.85) | 0.834 |
| Platelets (median (IQR)) | 211.00 (163.00, 274.00) | 217.00 (168.00, 266.50) | 0.971 |
| Red blood cells (median (IQR)) | 4.24 (3.85, 4.66) | 4.22 (3.77, 4.64) | 0.543 |
| Hemoglobin (median (IQR)) | 125.00 (108.00, 138.00) | 124.00 (107.50, 137.00) | 0.398 |
| Albumin (mean (SD)) | 39.80 (35.80, 42.70) | 39.70 (35.60, 42.70) | 0.569 |
| CRP (median (IQR)) | 3.84 (2.84, 18.10) | 3.51 (2.43, 16.80) | 0.62 |
| KPS score (median (IQR)) | 90.00 (80.00, 90.00) | 90.00 (80.00, 90.00) | 0.411 |
| PGSGA score (median (IQR)) | 6.00 (3.00, 9.00) | 5.00 (2.00, 9.00) | 0.975 |
| Cachexia, yes, n (%) | 342 (37.7) | 153 (39.4) | 0.591 |
| Global quality of life score (median (IQR)) | 46.00 (42.50, 52.00) | 47.00 (43.00, 53.50) | 0.643 |
| Short-term outcome, yes, n (%) | 52 ( 5.7) | 15 ( 3.9) | 0.212 |
| Status, death, n (%) | 289 (31.8) | 119 (30.7) | 0.729 |
| Length of hospitalization (median (IQR)) | 9.00 (5.00, 15.00) | 9.00 (5.00, 14.00) | 0.895 |
| Hospitalization expenses(median (IQR)) | 17894.65 (9879.88, 42096.22) | 16958.70 (9513.00, 41464.35) | 0.488 |

**Table S11.** Comparative analysis of the discrimination of each inflammation-related markers for all-cause mortality in CRC in validation cohorts.

| Validation cohort A | | | |
| --- | --- | --- | --- |
| Discrimination Ability | C-statistic | Difference | p value |
| IBI | 0.685(0.653,0.716) | Ref |  |
| NC | 0.681(0.649,0.714) | -0.003(-0.044, 0.036) | 0.875 |
| CAR | 0.682(0.651,0.713) | -0.003(-0.044, 0.038) | 0.905 |
| LCR | 0.676(0.645,0.708) | -0.008(-0.051, 0.032) | 0.703 |
| PC | 0.669(0.637,0.701) | -0.015(-0.057, 0.026) | 0.476 |
| NAR | 0.642(0.607,0.677) | -0.043(-0.093, 0.007) | 0.097 |
| GPS | 0.635(0.604,0.667) | -0.049(-0.095, -0.002) | 0.038 |
| mGPS | 0.629(0.597,0.660) | -0.056(-0.103, -0.008) | 0.021 |
| NLR | 0.625(0.591,0.660) | -0.059(-0.111, -0.012) | 0.019 |
| SII | 0.617(0.582,0.651) | -0.068(-0.121, -0.018) | 0.010 |
| NP | 0.609(0.574,0.644) | -0.076(-0.131, -0.022) | 0.006 |
| LCS | 0.618(0.592,0.645) | -0.066(-0.117, -0.019) | 0.008 |
| PAR | 0.575(0.540,0.611) | -0.109(-0.167, -0.050) | <0.001 |
| LA | 0.576(0.539,0.613) | -0.108(-0.169, -0.048) | <0.001 |
| PLR | 0.560(0.523,0.597) | -0.125(-0.215, 0.039) | 0.054 |
| Validation cohort B | | | |
| IBI | 0.626(0.571,0.682) | Ref |  |
| NC | 0.619(0.561,0.677) | -0.008(-0.026, 0.011) | 0.420 |
| CAR | 0.611(0.551,0.670) | -0.016(-0.036, 0.006) | 0.143 |
| LCR | 0.615(0.559,0.671) | -0.011(-0.029, 0.006) | 0.214 |
| PC | 0.608(0.548,0.668) | -0.019(-0.045, 0.006) | 0.150 |
| NAR | 0.614(0.560,0.668) | -0.012(-0.060, 0.032) | 0.597 |
| GPS | 0.597(0.545,0.649) | -0.029(-0.067, 0.005) | 0.110 |
| mGPS | 0.602(0.552,0.652) | -0.024(-0.061, 0.010) | 0.175 |
| NLR | 0.607(0.555,0.658) | -0.019(-0.079, 0.049) | 0.554 |
| SII | 0.610(0.555,0.665) | -0.016(-0.063, 0.030) | 0.495 |
| NP | 0.602(0.547,0.658) | -0.024(-0.082, -0.030) | 0.397 |
| LCS | 0.567(0.519,0.615) | -0.060(-0.102, -0.015) | 0.007 |
| PAR | 0.582(0.523,0.640) | -0.045(-0.108, 0.015) | 0.154 |
| LA | 0.551(0.494,0.607) | -0.076(-0.135, -0.012) | 0.016 |
| PLR | 0.561(0.504,0.617) | -0.066(-0.128, -0.001) | 0.044 |

Table note: cNRI, continuous net reclassification improvement; IDI, integrated discrimination improvement; AGR, Albumin-Globulin ratio; KPS, Karnofsky Performance Status; MAC, mid-arm circumference; HGS, hand grip strength; PG-SGA, patient-generated subjective nutrition assessment.
